# Public support for harm reduction: A population survey of Canadian adults

**DOI:** 10.1371/journal.pone.0251860

**Published:** 2021-05-19

**Authors:** T. Cameron Wild, Jakob Koziel, Jalene Anderson-Baron, Mark Asbridge, Lynne Belle-Isle, Colleen Dell, Richard Elliott, Andrew Hathaway, Donald MacPherson, Keely McBride, Bernie Pauly, Carol Strike, Adam Galovan, Elaine Hyshka

**Affiliations:** 1 School of Public Health, University of Alberta, Edmonton, Alberta, Canada; 2 Department of Community Health and Epidemiology, Department of Emergency Medicine, Dalhousie University, Halifax, Nova Scotia, Canada; 3 Canadian Institute for Substance Use Research, University of Victoria, Victoria, British Columbia, Canada; 4 Department of Sociology, School of Public Health, University of Saskatchewan, Saskatoon, Saskatchewan, Canada; 5 Canadian HIV/AIDS Legal Network, Toronto, Ontario, Canada; 6 Department of Sociology and Anthropology, University of Guelph, Guelph, Ontario, Canada; 7 Canadian Drug Policy Coalition, Vancouver, British Columbia, Canada; 8 Alberta Health, Edmonton, Alberta, Canada; 9 Dalla Lana School of Public Health, University of Toronto, Toronto, Ontario, Canada; 10 Department of Human Ecology, University of Alberta, Edmonton, Alberta, Canada; Universidad Nacional de Educacion a Distancia, SPAIN

## Abstract

We described public views toward harm reduction among Canadian adults and tested a social exposure model predicting support for these contentious services, drawing on theories in the morality policy, intergroup relations, addiction, and media communication literatures. A quota sample of 4645 adults (18+ years), randomly drawn from an online research panel and stratified to match age and sex distributions of adults within and across Canadian provinces, was recruited in June 2018. Participants completed survey items assessing support for harm reduction for people who use drugs (PWUD) and for seven harm reduction interventions. Additional items assessed exposure to media coverage on harm reduction, and scales assessing stigma toward PWUD (α = .72), personal familiarity with PWUD (α = .84), and disease model beliefs about addiction (α = .79). Most (64%) Canadians supported harm reduction (provincial estimates = 60% - 73%). Five of seven interventions received majority support, including: outreach (79%), naloxone (72%), drug checking (70%), needle distribution (60%) and supervised drug consumption (55%). Low-threshold opioid agonist treatment and safe inhalation interventions received less support (49% and 44%). Our social exposure model, adjusted for respondent sex, household income, political views, and education, exhibited good fit and accounted for 17% of variance in public support for harm reduction. Personal familiarity with PWUD and disease model beliefs about addiction were directly associated with support (*β*s = .07 and -0.10, respectively), and indirectly influenced public support via stigmatized attitudes toward PWUD (*β*s = 0.01 and -0.01, respectively). Strategies to increase support for harm reduction could problematize certain disease model beliefs (e.g., “There are only two possibilities for an alcoholic or drug addict–permanent abstinence or death”) and creating opportunities to reduce social distance between PWUD, the public, and policy makers.

## Introduction

A substantial evidence base supports the population benefits and cost-effectiveness of harm reduction interventions (e.g., needle distribution, supervised drug consumption) for people who use drugs (PWUD) [1–3]. From an instrumental-rational perspective, such evidence should translate directly into policies that institutionalize harm reduction services as routine interventions in health systems. However, these services have been contentious and implementation of them continues to be haphazard [4–8].

The literature on *morality policy* is helpful for understanding this disconnection between evidence and haphazard implementation of harm reduction services [9, 10]. Scholars in this area propose that when decision makers must reconcile conflicting public values over the legitimacy of providing health or social services to target populations, they strategically downplay instrumental support in favor of policy designed to serve rhetorical, symbolic functions [11, 12]. This insight has informed the Canadian Harm Reduction Policy Project (CHARPP), a mixed-method, multiple case study drawing on four data sources (policy documents, informant interviews, media coverage, and a national public opinion survey) to analyze how policies governing harm reduction services are positioned within and across the Canadian provinces and territories. In previous work, CHARPP analyzed harm reduction policies written by governments and health authorities. Two studies confirmed that policies were largely produced for rhetorical rather than instrumental purposes, as revealed in documents that avoided clear governance statements (e.g., timelines, funding arrangements, governmental endorsements, references to legislation), and failed to name or support specific harm reduction interventions or key international tenets of harm reduction (i.e., abstaining from substance use is not required to receive health services, stigma and discrimination are often faced by substance users, PWUD should be involved in policy making) [13, 14]. A complementary CHARPP study interviewed governmental officials, health system leaders, and people with lived/living experience, confirming that Canadian policies offer weak instrumental support for harm reduction. Policy actors expressed ambivalence about the utility of formal policy and described how they adopted pragmatic strategies to support harm reduction services in morality policy environments [15]. Finally, CHARPP analyses of 17 years of Canadian newspaper coverage concluded that harm reduction was rarely portrayed negatively or from a criminal perspective. Volume of coverage tracked major events (e.g., Canada’s opioid emergency, legal challenges to Vancouver’s safe injection programming) but dramatically overemphasized supervised drug consumption and naloxone programs at the expense of other harm reduction services. This limited sense of ‘newsworthiness’ may have perpetuated a morality policy environment for harm reduction by reducing public awareness of the full range of evidence-supported harm reduction services that could benefit PWUD [16].

Public acceptability is of course a key consideration in developing policy frameworks for harm reduction services and is the focus of the present study. Canadian and Australian research has documented substantial public support for a variety of harm reduction services, including supervised injection programs [17–22], needle distribution [19, 23–26], and safer inhalation programs [25, 27]. However, research is limited because public opinion has been described regionally within those countries, and typically for select, newsworthy harm reduction services rather than the full spectrum of evidence-supported interventions. Nationally representative surveys of US adults revealed that most respondents were either neutral or opposed toward needle distribution and supervised injection programs [19, 23, 26], but no similar research has comprehensively described national and regional public opinions toward harm reduction in Canada.

**Fig 1:**
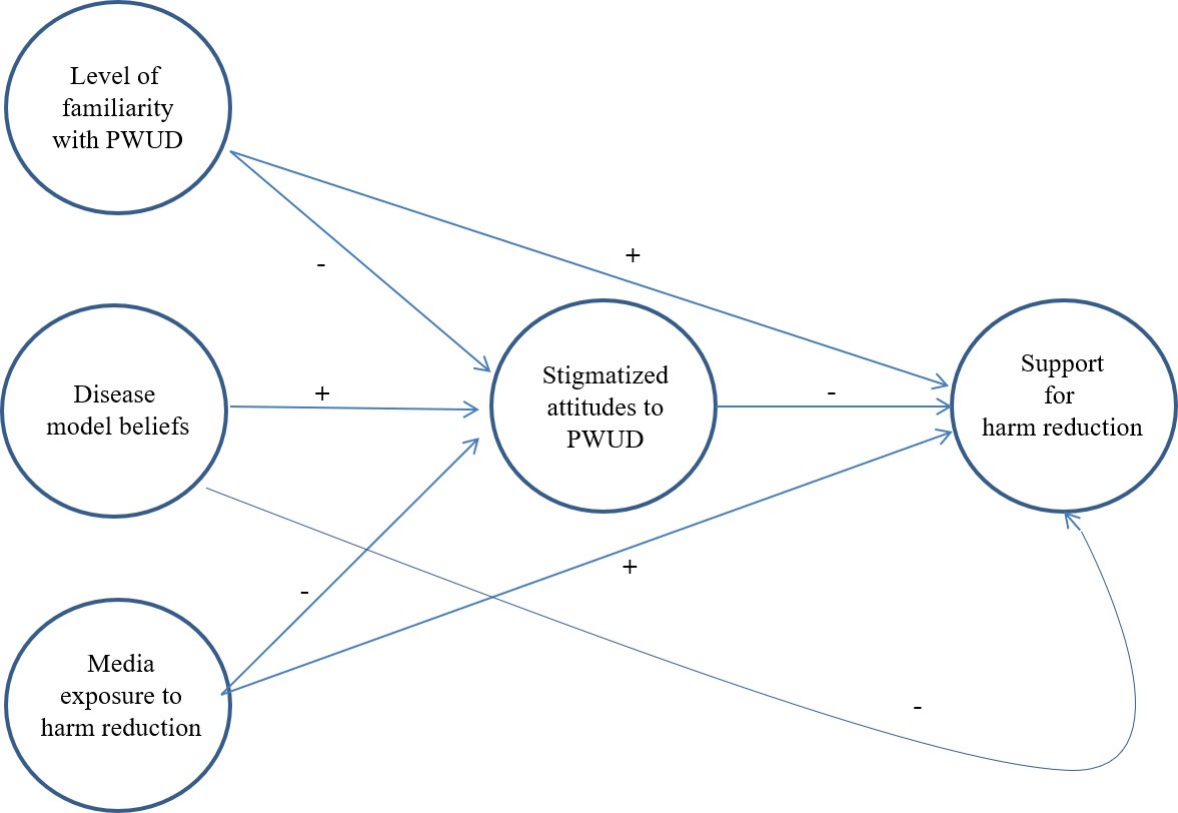
Hypothesized social exposure model of public support for harm reduction.

The literature on public views toward harm reduction is also limited because few studies have examined correlates of public support. Extant research has emphasized sociodemographic correlates, revealing that liberal political views, higher educational attainment, and income are positively associated with public support for harm reduction [17, 19, 23, 25]. However, little work has tested plausible theoretical models that could inform intervention strategies to modify public opinion. To address this gap, we propose a social exposure model depicted in Fig 1 wherein four constructs influence public support for harm reduction services, drawing on theories in the morality policy, intergroup relations, addiction, and media communication literatures.

Our model proposes that *stigmatized attitudes toward PWUD* are a proximal determinant of public views on harm reduction services. This prediction reflects a key tenet of morality policy studies [9], namely that emphasizing the ostensibly immoral, deviant behaviors of a target population (PWUD) renders evidence on intervention effectiveness irrelevant, thus calling into question the legitimacy of offering health services to them. When opponents of harm reduction services adopt this position, they essentially view PWUD as a stigmatized outgroup, unworthy of receiving effective interventions. Thus, we hypothesize that stigmatized attitudes toward PWUD will be inversely associated with public support for harm reduction–a prediction that has been supported in two recent US studies [19, 23]. Stigmatized attitudes may in turn be influenced by *level of familiarity with PWUD*. The intergroup relations literature has provided empirical support for the contact hypothesis, according to which greater exposure to and familiarity with outgroups facilitates empathic personal attitudes toward outgroup members [28–30]. Thus, we hypothesize that personal familiarity with PWUD will be inversely associated with stigmatized attitudes and positively associated with support for harm reduction.

Although not previously investigated, beliefs about addiction, and in particular, *disease model beliefs*, may also be relevant for understanding stigma toward PWUD and public views toward harm reduction services. Harm reduction programs are contentious in part because PWUD do not need to abstain from substance use in order to receive them. This key tenet of harm reduction problematizes disease model thinking, according to which drug and alcohol dependence is chronic relapsing brain disorder [31] that can only be mitigated by complete abstinence [32]. From this perspective, harm reduction services are problematic because they ‘enable’ substance use and perpetuate the disease. Thus, we hypothesize that disease model beliefs will be positively associated with stigma toward PWUD and inversely associated with support for harm reduction. Finally, cultivation theory proposes that *exposure to media* shapes peoples’ views on policy responses to contentious social issues [33]. For example, exposure to violent media programming is positively associated with beliefs that one will become a victim of violence and also with support for punitive and retributive legislation [34]. Although not previously investigated, we hypothesize that exposure to media reporting on harm reduction will be inversely associated with stigmatized attitudes toward PWUD and positively associated with support for harm reduction.

### Objectives

Our *first objective* was to describe the nature and extent of public support for harm reduction as a broad approach to substance use in Canada, and also in relation to seven specific harm reduction interventions: supervised consumption, syringe distribution, naloxone, low threshold opioid treatment, community outreach, drug checking, and safer inhalation services. Our *second objective* was to test our social exposure model. Specifically, we sought to answer three research questions implied by our model, including: (a) whether stigmatized attitudes toward PWUI are inversely associated with public support for harm reduction, (b) whether personal familiarity with PWUD, disease model beliefs about addiction, and exposure to media coverage on harm reduction are positively, inversely, and positively associated with support for harm reduction, respectively, and (c) whether these distal social exposure variables operate indirectly to influence public support for harm reduction, via stigmatized attitudes toward PWUD.

## Materials and methods

### Sample and procedure

Participants were recruited from an online research panel (Ipsos Canadian Online Panel), and the survey methods were designed to produce generalizable estimates of public opinion toward harm reduction at both national and provincial levels using a two-phased sampling procedure. In phase 1, randomly-drawn Canadian adult panel members were invited to participate until a quota sample of 2002 respondents matching the age and sex distributions of Canadian adults (18+ years) residing in each major region of Canada (i.e., BC, Alberta, Prairie region [Saskatchewan/Manitoba], Ontario, Quebec, Atlantic region [Nova Scotia, New Brunswick, Prince Edward Island, Newfoundland] was obtained. In phase 2, a booster sample of 2643 respondents was recruited to oversample individual provinces, i.e., to provide representative estimates for each Canadian province; sampling proceeded until a quota sample matching the age and sex distributions of Canadian adults residing in each of the 10 Canadian provinces was recruited. National and provincial quotas within age and sex strata were based on the 2016 Canadian Census. Canadians residing in the territories (Northwest Territories; Nunavut; Yukon) were not invited to participate in either sampling phase. The final sample included 4645 adults, 18 years of age or older. Analyses of the phase 1 subsample provided nationally representative estimates; analyses of the total sample provided provincially representative estimates. In order to provide accurate parameter estimates and to avoid errors in calculating variances, survey weights were converted to normalized (relative) weights for each respondent. Relative weights were calculated by dividing the survey weight of a respondent by the mean of all survey weights [35, 36]. The sum of the survey and relative weights in the nationally (*n* = 2002) and provincially (*N* = 4645) representative datasets each equaled their respective sample sizes and as such the original sample weights were used for subsequent analyses. Sample characteristics are provided in Table 1.

**Table 1:** Sociodemographic characteristic of the sample.

Participant characteristics (%)	Unweighted N (%)		Weighted N (%)	
	Subsample (nationally representative; *n* = 2,002)	Total sample (provincially representative; *N* = 4,645)	Subsample (nationally representative; *n* = 2,002)	Total sample (provincially representative; *N* = 4,645)
**Sex**				
Male	901 (45.0)	2194 (47.2)	972 (48.6)	2256 (48.6)
Female	1101 (55.0)	2451 (52.8)	1030 (51.4)	2389 (51.4)
**Age**				
18–24	131 (6.5)	313 (6.7)	129 (6.4)	327 (7.0)
25–34	414 (20.7)	892 (19.2)	419 (20.9)	912 (19.6)
35–44	339 (16.9)	669 (14.4)	315 (15.7)	669 (14.4)
45–54	394 (19.7)	899 (19.4)	366 (18.3)	899 (19.4)
55–64	441 (22.0)	1000 (21.5)	469 (23.4)	985 (21.2)
65–74	240 (12.0)	715 (15.4)	257 (12.8)	701 (15.1)
75–84	40 (2.0)	140 (3.0)	43 (2.1)	136 (2.9)
85–94	2 (0.1)	15 (0.3)	2 (0.1)	15 (0.3)
95+	1 (0.1)	2 (0.04)	1 (0.1)	2 (0.04)
**Province**				
British Columbia	273 (13.6)	502 (10.8)	272 (13.6)	502 (10.8)
Alberta	225 (11.2)	501 (10.8)	224 (11.2)	501 (10.8)
Saskatchewan	131 (6.5)	400 (8.6)	130 (6.5)	400 (8.6)
Manitoba		400 (8.6)		400 (8.6)
Ontario	768 (38.4)	800 (17.2)	769 (38.4)	800 (17.2)
Quebec	468 (23.4)	501 (10.8)	470 (23.5)	501 (10.8)
Nova Scotia	137 (6.8)	400 (8.6)	136 (6.8)	400 (8.6)
New Brunswick		400 (8.6)		400 (8.6)
Prince Edward Island		341 (7.3)		341 (7.3)
Newfoundland		400 (8.6)		400 (8.6)
**Political Views**				
Very conservative	115 (5.7)	270 (5.8)	116 (5.8)	270 (5.8)
Mostly conservative	336 (16.8)	816 (17.6)	338 (16.9)	816 (17.6)
Equal	452 (22.6)	1086 (23.4)	456 (22.8)	1089 (23.4)
Mostly liberal	494 (24.7)	1070 (23.0)	502 (25.1)	1074 (23.1)
Very liberal	186 (9.3)	392 (8.4)	186 (9.3)	394 (8.5)
No political views	291 (14.5)	666 (14.3)	282 (14.1)	663 (14.3)
Prefer not to say	128 (6.4)	345 (7.4)	121 (6.0)	339 (7.3)
**Annual household income**				
< $50,000	681(34.0)	1544 (33.2)	680 (34.0)	1540 (33.2)
$50,000 - $100,000	749 (37.4)	1714 (36.9)	747 (37.3)	1720 (37.0)
>$100,000	382 (19.1)	853 (18.4)	388 (19.4)	859 (18.5)
Prefer not to say	190 (9.5)	534 (11.5)	187 (9.3)	527 (11.3)
**Education**				
High school diploma	476 (23.8)	1135 (24.4)	473 (23.6)	1135 (24.4)
College/technical school	702 (35.1)	1737 (37.4)	700(35.0)	1725 (37.1)
University graduate	824 (41.2)	1773 (38.2)	830 (41.5)	1785 (38.4)
**Location of residence**				
Rural	440 (22.0)	1377 (29.6)	432 (21.6)	1368 (29.5)
Urban	1562 (78.0)	3268 (70.4)	1570 (78.4)	3277 (70.5)

In both sampling phases, panel members received email invitations which included a personal identification number along with a URL link to an information letter/informed consent procedure (S1 File). Consenting participants completed the survey online at their convenience from May 31 –June 25, 2018 and had the ability to leave the survey and complete it at another time [36]. In order to maximize participation and minimize nonresponse bias, email reminders were sent approximately three days following the initial invitation, and an incentive (points allocated toward quarterly prize draws for panel members) was provided to all respondents who completed the survey.

### Measures

Items and scales used in the present study were drawn from four survey modules: (1) opinions on national and provincial responses to substance use, (2) opinions on harm reduction as an approach to substance use and seven specific harm reduction interventions, (3) personal experiences with, and attitudes toward substance use and addictions, and (4) sociodemographics (S1 File).

#### General support for harm reduction

Three survey items assessed public views toward harm reduction as an approach to substance use. These questions were preceded by a definition of harm reduction, which was neutrally framed to acknowledge supportive and opposing positions (i.e., “*Harm reduction refers to public health programs that reduce the harms related to drug use*, *without requiring people to stop using substances*. *An example would be providing supervised injection sites to people who inject drugs so that they can use drugs more safely*. *There are lots of different opinions about harm reduction*. *Supporters think these programs can significantly reduce death and the transmission of disease among people who use drugs*, *and that these programs can bring them into contact with health and social services that could help in their recovery*. *Opponents argue that harm reduction programs encourage crime and drug use and should not be offered*.”). Respondents rated their level of personal support for harm reduction (strongly oppose, oppose, don’t know/no opinion, support, strongly support, prefer not to say). Responses were recoded as 1 = oppose (i.e., strongly oppose or oppose), 2 = don’t know/no opinion, and 3 = support (i.e., support or strongly support); respondents who endorsed ‘prefer not to say’ were recoded as missing and removed from weighted parameter estimates regarding that particular question. Two questions assessed support for government action on harm reduction (i.e., “*My federal* [and in a separate question, *provincial*] *government should provide more financial and other support for harm reduction services*”), each followed by six responses (strongly disagree, disagree, don’t know/no opinion, agree, strongly agree, prefer not to say). Responses were recoded as 1 = disagree (i.e., strongly disagree or disagree), 2 = don’t know/no opinion, and 3 = support (i.e., agree or strongly agree); respondents who endorsed ‘prefer not to say’ were recoded as missing and removed from weighted parameter estimates regarding that particular question.

#### Support for specific harm reduction interventions

Participants provided their views on seven harm reduction services: supervised drug consumption, syringe distribution, naloxone, low threshold opioid treatment (i.e., opioid agonist medications delivered without imposing abstinence as a condition for access), community outreach, drug checking, and safer inhalation kits. Each service was defined for respondents, followed by one item assessing support (strongly oppose, oppose, don’t know/no opinion, support, strongly support, prefer not to say). Responses were recoded as 1 = oppose (i.e., strongly oppose or oppose), 2 = don’t know/no opinion, and 3 = support (i.e., support or strongly support); respondents who endorsed ‘prefer not to say’ were recoded as missing and removed from weighted parameter estimates regarding that particular question.

#### Stigmatized attitudes toward PWUD

Stigma was assessed using four social distance items (α = .72 in the present sample) modified from the World Psychiatric Association’s Schizophrenia: *Open the Door project* [37]: (1) would you be afraid to talk to someone who has a substance use problem? (2) would you be upset or disturbed to be in the same room with someone who has a substance use problem? (3) would you make friends with someone who has a substance use problem? and (4) would you feel embarrassed or ashamed if your friends knew that someone in your family has a substance use problem? Each item was accompanied by a 6-point response scale (definitely not, probably not, not sure/don’t know, probably, definitely, prefer not to say); respondents who endorsed ‘prefer not to say’ were recoded as missing for model testing. The third stigma question was reverse-coded so that higher scores were indicative of stronger stigmatized attitudes.

#### Level of familiarity with PWUD

Respondents completed the level of familiarity (LOF) scale [38, 39], modified in this study to assess how familiar respondents were with people who have substance use problems (α = .84 in the present sample). The scale includes 11 dichotomous items ranging from no familiarity (e.g., “I have never observed a person that I was aware had a substance use problem” [LOF score = 1] to maximum familiarity (e.g., “I have a substance use problem” [LOF score = 11]), with additional items assessing moderate familiarity (e.g., “I have watched a documentary on television about substance use problems” [LOF score = 4). Respondents indicated whether each statement was true or false for them, and an overall LOF score was assigned based on respondents’ highest level of familiarity. For example, if a respondent indicated that they watched a documentary about persons with a substance use problem (LOF score = 4) and also indicated that they have a relative who has a substance use problem (LOF score = 9) that respondent would receive a LOF score of 9. Respondents who endorsed ‘none of the above’ were recoded as missing.

#### Disease model beliefs

Respondents completed the 7-item disease model beliefs subscale from the Short Understanding of Substance Abuse Scales (α = .79 in the present sample) [32, 40]. Items assessed agreement with views that addiction is a chronic relapsing disorder that can only be ameliorated with abstinence (e.g., “There are only two possibilities for an alcoholic or drug addict–permanent abstinence or death”; “Once a person is an alcoholic or an addict, he or she will always be an alcoholic or an addict”). Response options were recorded using a 6-point response scale (strongly disagree, disagree, don’t know/no opinion, agree, strongly agree, prefer not to say); respondents who endorsed ‘prefer not to say’ were recoded as missing for model testing.

#### Media exposure to harm reduction

Two survey items developed for this study assessed respondents’ exposure to harm reduction via the media. Specifically, participants indicated whether they had ever seen or heard media coverage of harm reduction (yes, no), and media coverage featuring bereaved mothers who had a child die from a fatal drug overdose (yes, no).

#### Sociodemographics

Participants’ sex (male, female), age, and educational attainment (high school completion or less, technical school/college diploma, university degree(s)) were collected as part of the survey sampling procedures. In addition, a single survey item asked participants to identify their political views (i.e., very liberal, mostly liberal, equally liberal and conservative, mostly conservative, very conservative, I don’t have any political views, prefer not to say), annual household income (< $50,000 CDN, $50,000 - $100,000, > $100,000), and whether they lived in a rural or urban area.

### Analyses

#### Objective 1

We calculated proportions of the sample expressing support (i.e., agree/strongly agree; support/strongly support) for harm reduction as a general response to substance use and for seven specific interventions. To account for the complex survey design, standard errors and 95% confidence intervals for weighted proportions were estimated using a set of 500 bootstrap weights computed using MPlus version 8.4 [36]. The survey design stratified respondents by age and sex within each province or region; a stratification variable was created in which each respondent was placed into one of six strata based on age (18–34, 35–54, 55–100 years) and sex (males, females). The primary sampling units (PSUs) for weighted estimates for the phase 1 sample were region (Alberta, Atlantic Provinces, British Columbia, Ontario, Quebec, Saskatchewan and Manitoba) whereas the PSUs for the total, provincially representative dataset, were the ten Canadian provinces. Province was defined as a cluster variable for the provincial analyses of the entire dataset, while region was defined as a cluster variable for the national analyses using the representative Canadian subsample. These clustering and stratification variables were used to create 500 bootstrap weights for the entire (provincially representative) dataset and the nationally representative Canadian subsample and to estimate variances. Once standard errors were produced using 500 bootstrap weights, a coefficient of variation was calculated for each weighted proportion by dividing the standard error by the weighted proportion to derive the sampling variability percentage. Using Statistics Canada criteria, parameter estimates exhibiting sampling variability of less than 16.6% were considered acceptable while estimates with variability greater than 16.5% but less than 33.3% were classified as moderate, and were annotated with an ‘interpret with caution’ descriptor. All weighted estimates of population proportions had sample sizes of more than thirty individuals [41].

#### Objective 2

MPlus version 8.4 and R version 3.6.1 were used to evaluate the hypothesized model predicting public support for harm reduction depicted in Fig 1. First, in order to describe the relationship between latent variables presented in Fig 1 and their indicators, a measurement model was estimated [42]. Constructs that were assessed using pre-existing scales drawn from the literature (i.e., LOF scale, disease model beliefs scale) were treated as single composite indicator scale scores, while constructs assessed using new indicator items developed for this study (i.e., media exposure to harm reduction, support for harm reduction) were treated as latent variables. Due to the borderline alpha coefficient observed for its 4-item composite scale, we also treated the stigma construct as a latent variable in our analyses and investigated the measurement structure of those items.

Factor loadings and correlations between indicators and latent variables were assessed these each latent constructs, followed by confirmatory factor analysis. Second, a structural equation model (SEM) was estimated to evaluate direct and indirect effects of the constructs depicted in Fig 1. Results for this model were compared to a second SEM that included four covariates (political views, income, education, and respondent sex) to produce covariate-adjusted estimates.

A weighted least squares means and variance adjusted method was utilized to accommodate missing data and categorical variables in evaluating both the measurement and structural models. Maximum likelihood estimation was used to calculate the correlations among continuous variables and account for missing data. Shapiro-Wilk tests of normality were conducted on each independent variable to assess whether they were normally distributed. Model fit was evaluated using several indices (i.e., Root Mean Square Error of Approximation [RMSEA], Comparative Fit Index [CFI], Standardized Root Mean Square Residual [SRMSR]; Chi square [χ^2^]).

### Ethics statement

The study procedures and measures were approved by the University of Alberta Health Research Ethics Board. Ethics approval was obtained from the University of Alberta under the title “National Survey of Public Opinion on Harm Reduction Services and Drug Use”, study ID: Pro00080911.

## Results

### Objective 1: Describing public support for harm reduction

**Fig 2:**
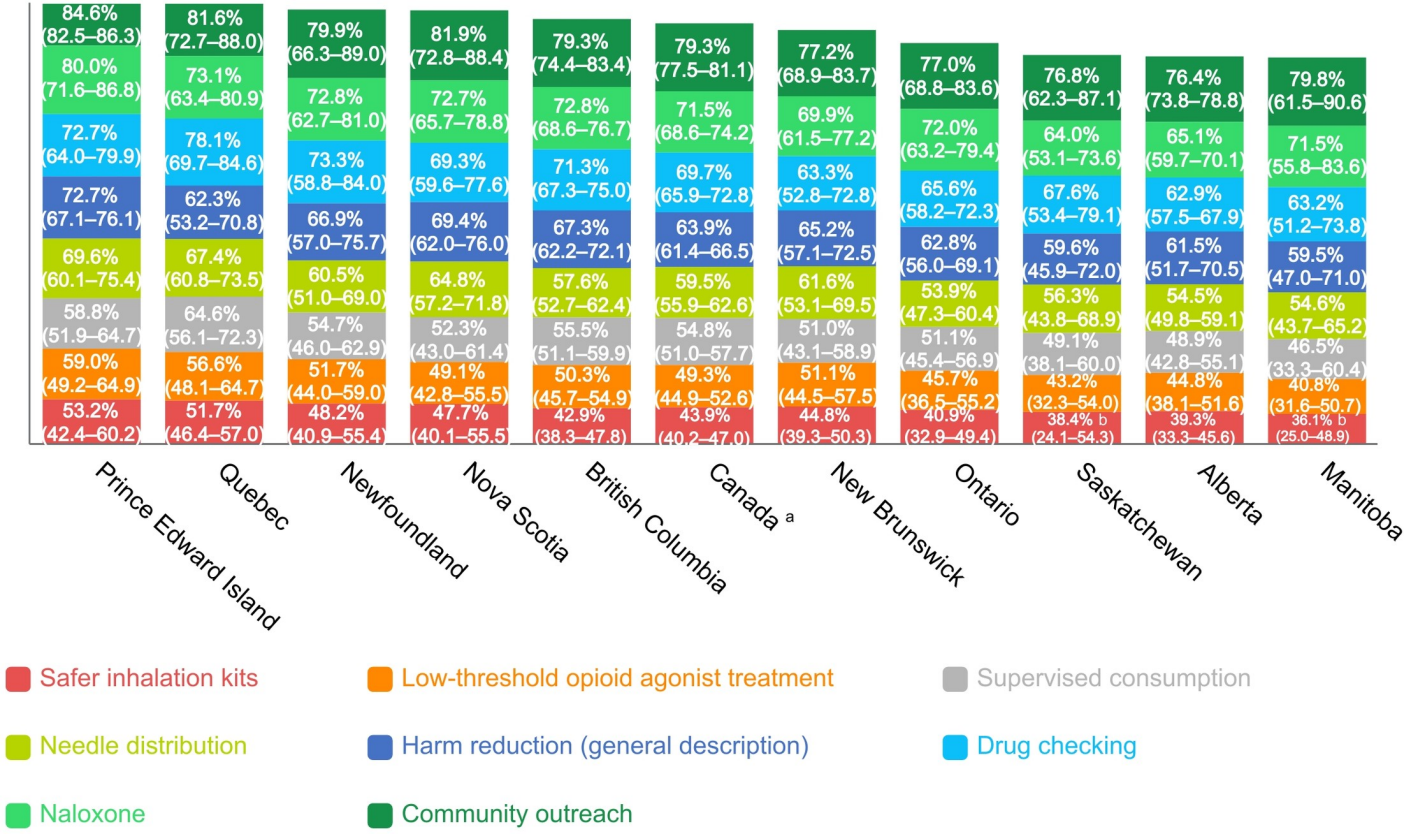
Proportion of Canadians supporting or strongly supporting harm reduction, 2018. ^a^ Canadian estimates calculated on the nationally representative subsample (*n* = 2002). ^b^ Moderate sampling variability (>16.6%), interpret with caution.

Canadians were generally supportive (64%) of harm reduction as an approach to substance use (Fig 2). Public support varied across different harm reduction services, with more than three quarters supporting community outreach (79%) and over 70% supporting naloxone distribution (72%) and drug checking interventions (70%) (Fig 2). Needle distribution (60%) and supervised drug consumption programs (55%) received lesser, though still majority, support (Fig 2). Low-threshold opioid agonist treatment (49%) and safer inhalation kits (44%) received the least amount of support among Canadians at the national level (Fig 2).

Public views at the provincial level revealed some regional diversity. Specifically, respondents residing in the Atlantic region of Canada (New Brunswick, Nova Scotia, Newfoundland and Labrador, Prince Edward Island) and British Columbia reported most support for harm reduction (as a general approach, and among the 7 specific services measured) compared to other provinces and regions, while central Canadians (Ontario, Quebec) tended to view harm reduction more moderately. Respondents living in the Canadian prairie provinces (Alberta, Saskatchewan, and Manitoba) reported the lowest levels of support for harm reduction compared to other regions.

### Objective 2: Evaluating a social exposure model of public support for harm reduction

#### Latent constructs, indicators, and descriptive statistics

Fig 1 includes five latent constructs. Level of familiarity with PWUD was assessed using a single continuous indicator variable, LOF scores. Disease model beliefs were assessed using a single continuous indicator variable, disease model subscale scores. The latent media exposure to harm reduction construct was assessed using two categorical items: whether or not respondents reported ever seeing or hearing media coverage (a) featuring harm reduction and (b) bereaved mothers who had a child die from a drug overdose. Stigmatized attitudes toward PWUD was treated as a latent construct measured using four continuous indicator variables drawn from this module of the survey. Shapiro-Wilk tests of normality revealed that each study variable was non-normally distributed; however, estimates of kurtosis and skewness for these variables (Table 2) indicated that these data were within the acceptable non-normal cut-off points recommended by Kline [43]. Our latent outcome construct, support for harm reduction, was assessed using three continuous indicator variables: overall support for harm reduction, support for increased federal investment in harm reduction, and support for increased provincial investment in harm reduction.

**Table 2:** Descriptive statistics and correlations among continuous study variables (N = 4645).

Variable	1	2	3	4	5	6	7	8	9	10	11	12	13
1.LOF	--												
2.Disease	-.03	--											
3.Stig1	-.19**	.14**	--										
4.Stig2	-.17**	.21**	.63**	--									
5.Stig3	-.17**	.14**	.23**	.31**	--								
6.Stig4	-.17**	.14**	.47**	.50**	.20**	--							
7.HRsuppt	.07**	-.18**	-.11**	-.13**	-.24**	-.09**	--						
8.ProvHR	.09**	-.14**	-.06**	-.09**	-.27**	-.07**	.75**	--					
9.FedHR	.09**	-.14**	-.06**	-.09**	-.26**	-.06**	.75**	.91**	--				
10.Age	-.09**	.26**	-.15**	-.09**	.00	-.10**	-.10**	-.08**	-.06**	--			
11.Politics	-.05**	.18**	.10**	.11**	.12**	.10**	-.33**	-.34**	-.32**	.11**	--		
12.Income	.02	-.06**	.03	.01	.03	.08**	-.01	-.04*	-.04*	-.06**	0.04*	--	
13.Education	-.02	-.10**	.05**	.03	-.03	.09**	.09**	.05**	.06**	-.09**	-.10**	0.29**	--
*Mean (M)*	6.22	22.90	2.37	2.55	2.98	2.66	3.64	3.46	3.55	48.23	2.86	1.83	2.14
*Median*	6	23	2	2	3	2	4	4	4	50	3	2	2
*Standard deviation (SD)*	2.68	5.46	1.16	1.21	1.08	1.25	1.23	1.27	1.27	16.02	1.11	0.74	0.78
*Skewness*	.01	-0.18	0.64	0/49	0.07	0.30	-.68	-.46	-.58	-.03	0.13	0.28	-0.25
*Kurtosis*	-1.31	-0.16	-0.52	-0.80	-0.70	-1.05	-.58	-.92	-.81	-1.03	-0.76	-1.16	-1.32

^a^ LOF = level of familiarity scale. Disease = disease model beliefs. Stig = stigma items. HRsupport = rated support for harm reduction (general description). ProvHR and FedHR = rated support for increasing resources to harm reduction services.

* p < 0.05;

** p < 0.01.

Correlations among continuous indicator variables along with their accompanying descriptive statistics are presented in Table 2. The average age of participants was 48.2 years (SD = 16.0). Respondents were slightly positive toward harm reduction on the response scale (*M* = 3.6, SD = 1.2) and also on whether the federal (*M* = 3.6, SD = 1.3) or provincial governments (*M* = 3.5, SD = 1.3) should increase financial and other supports regarding harm reduction. For the predictor variables, respondents reported moderate LOF with people experiencing substance use problems (*M* LOF score = 6.2, SD = 2.7), and in general, the sample held low stigmatizing attitudes toward PWUD (e.g., most indicated that they had little problem talking to someone with a substance use problem [item *M* = 2.4, SD = 1.2] or being in the same room with someone with a substance use problem [item *M* = 2.6, SD = 1.2]. Most respondents (57.3%) had seen media coverage regarding harm reduction as well as coverage featuring mothers whose children had died from a fatal drug overdose (58.3%). As shown in Table 2, variables ProvHR1 and FedHR1 were highly correlated with one another (> 0.9) while the remaining inter-item correlations were less than 0.85 [43].

We also compared median scores on our three indicators of support for harm reduction (overall support for harm reduction, support for increased federal investment in harm reduction, and support for increased provincial investment in harm reduction) across four sociodemographic covariates: annual household income, education, political views, and respondent sex. Median levels of support for harm reduction were consistent (median = 4; support) across all covariates except for political affiliation, where we observed differences in median levels of support were observed such that respondents who identified with ‘very’ and ‘mostly’ conservative political views reported lower median levels of support toward harm reduction (median = 3; don’t know/no opinion) compared to those who endorsed more liberal political affiliations.

#### Measurement model

The latent constructs were correlated with each other (*ps* ranged from 0.05 to 0.001; standardized values ranged from 0.06 to—0.18). A confirmatory factor analysis revealed that the standardized factor loadings for the stigma toward PWUD (values ranged from 0.41 to 0.81), support for harm reduction (values ranged from 0.84 to 0.93), and exposure to media coverage on harm reduction (values ranged from 0.59 to 0.65) latent variables in the hypothesized model were acceptable to high with the exception of the third stigma item (0.41) [44]. The low factor loading of the third stigma item (would you make friends with someone who has a substance use problem?) suggests that it was a weaker indicator of the stigma construct, and was dropped from the model [44]. Factor variance for the media exposure construct was set to 1 with the loadings freely estimated [43]. An initial confirmatory factor analysis for the hypothesized measurement model indicated poor global model fit (RMSEA = 0.083; CFI = 0.787; SRMSR = 0.052, χ^2^ = 798.437, p < 0.001, *df* = 24, *N* = 4645). The model was then re-specified by removing the third scale item from the stigma latent construct, given its low factor loading. A second confirmatory factor analysis indicated that the overidentified model exhibited good global fit (RMSEA = 0.026; CFI = 0.982; SRMSR = 0.016, χ^2^ = 70.248, p < 0.001, *df* = 17; *N* = 4645). Closer inspection of local fit also revealed that the model had good local fit while all factor loadings were considered acceptable to high and statistically significant (p < 0.001) [43, 44].

#### Structural model

**Fig 3:**
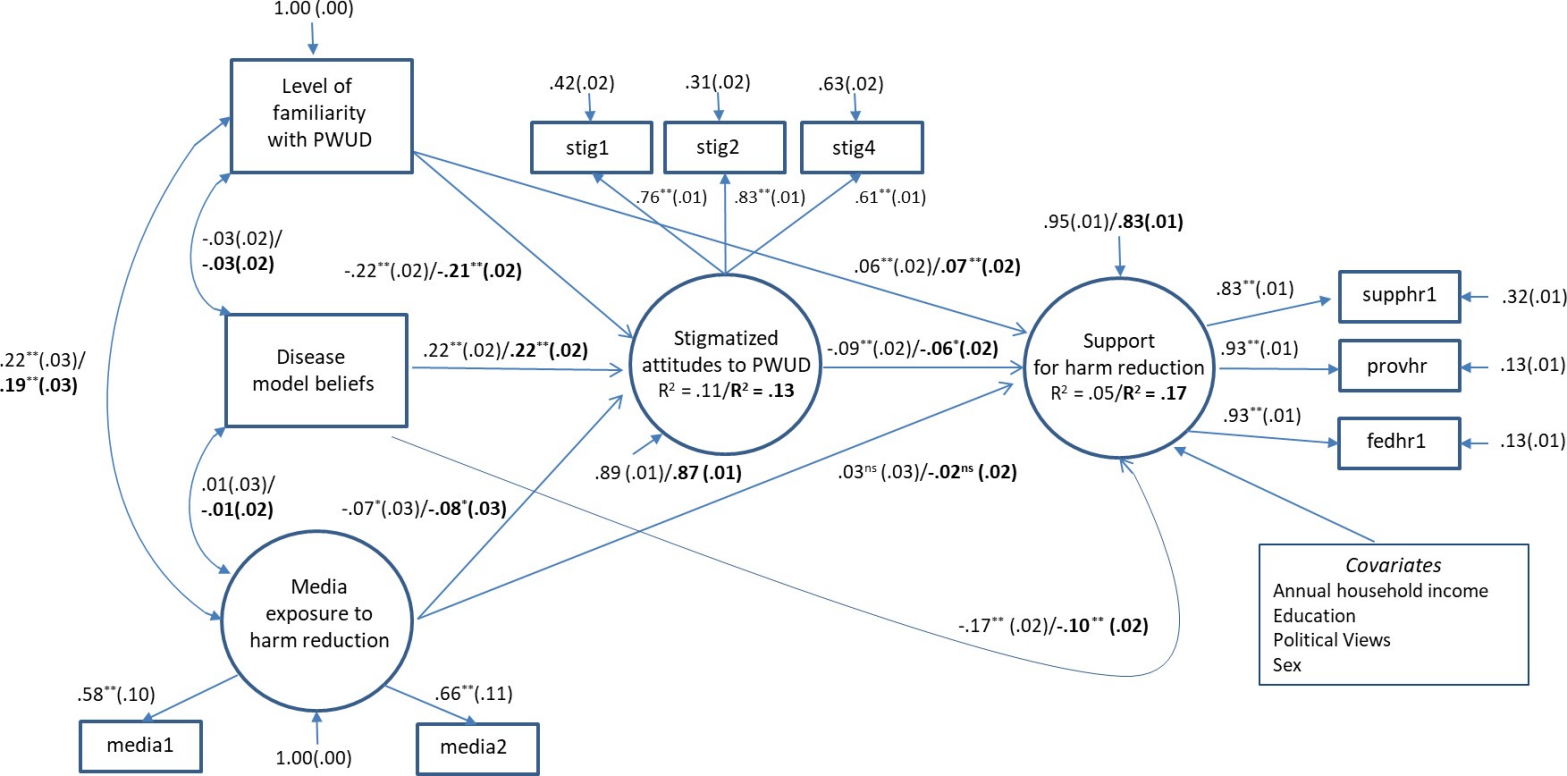
Final measurement and full structural model. Values are standardized coefficients (*β*) while the values in brackets are their corresbonding standard errors (SE); *p < .05; **p < .001; Unadjusted Model Fit (RMSEA = 0.022; CFI = 0.984; SRMSR = 0.015; χ^2^ = 85.937; p < 0.001, *df* = 26; *N* = 4645) / Adjusted Model Fit (RMSEA = 0.028; CFI = 0.968; SRMSR = 0.017; χ^2^ = 211.227; p < 0.001; *df* = 46; *N* = 4645).

A structural equation model, incorporating the survey weights, was fit in order to examine indirect and direct effects of level of familiarity, disease model beliefs, exposure to harm reduction media, and stigmatized attitudes toward PWUD on public support of harm reduction (Fig 3). Each path was tested to see whether it was nonzero and if so, whether the valence of the observed association confirmed theoretical predictions (Fig 3). Results from this SEM were compared to a second SEM that included sociodemographic covariates to obtain adjusted estimates (Table 3). In both analyses, data were weighted by age and sex.

**Table 3:** Indirect, direct, and total effects for the unadjusted and adjusted models.

Variables	Unadjusted model			Adjusted model		
	Indirect effects (SE)	Direct effects (SE)	Total effects (SE)	Indirect effects (SE)	Direct effects (SE)	Total effects (SE)
*Effects on stigmatized attitudes*						
Level of familiarity (LOF)	n/a	-0.216** (0.018)	-0.216** (0.018)	n/a	-0.212** (0.017)	-0.212** (0.017)
Disease model beliefs	n/a	0.216** (0.017)	0.216** (0.017)	n/a	0.215** (0.017)	0.215** (0.017)
Media exposure	n/a	-0.072* (0.027)	-0.072* (0.027)	n/a	-0.078* (0.026)	-0.078* (0.026)
*Effects on support for harm reduction*						
Level of familiarity (LOF)	0.019** (0.005)	0.063** (0.018)	0.081** (0.017)	0.012* (0.004)	0.067** (0.016)	0.080** (0.016)
Disease model beliefs	-0.019** (0.004)	-0.165** (0.018)	-0.184** (0.017)	-0.012* (0.004)	-0.103** (0.017)	-0.115** (0.017)
Media exposure	0.006* (0.003)	0.034 (0.028)	0.040 (0.028)	0.004* (0.002)	-0.019 (0.024)	-0.014 (0.024)
Stigmatized attitudes	n/a	-0.086** (0.019)	-0.086** (0.019)	n/a	-0.057* (0.019)	-0.057* (0.019)

Values are standardized coefficients;

* p *<* .05;

** p *<* .001.

The final weighted unadjusted structural model (Fig 3) exhibited good global model fit (RMSEA = 0.022; CFI = 0.984; SRMSR = 0.015; χ^2^ = 85.937; p < 0.001, *df* = 26; *N* = 4645) and local fit. The adjusted weighted structural model (Fig 3) also had good global model fit (RMSEA = 0.028; CFI = 0.968; SRMSR = 0.017; χ^2^ = 211.227; p < 0.001; *df* = 46; *N* = 4645) as well as good local fit. In order to estimate both the indirect and total effects using missing data, 1,000 bootstrap samples were estimated. Bias-corrected significance levels for all effects did not vary from the original unadjusted and adjusted models that did not use bootstrapping. The final unadjusted model explained over 11% of observed variance regarding stigmatized attitudes and 5% of observed variance regarding public support for harm reduction, while the adjusted model explained 13% of observed variance regarding stigmatized attitudes and 17% of observed variance regarding public support for harm reduction (Fig 3).

Table 3 presents indirect, direct, and total effects of the study variables on stigma and support for harm reduction. Inspection of direct effects in the structural model indicated that disease model belief about addiction were positively associated with stigmatized attitudes toward PWUD in the unadjusted and adjusted models (*β* = 0.22, p < 0.001) while, level of familiarity with PWUD was inversely associated with stigmatizing beliefs toward PWUD in both the unadjusted and adjusted models (*β* = -0.22, p < 0.001).

Additionally, media exposure to harm reduction was inversely associated with stigmatized attitudes in both the unadjusted (*β* = -0.07, p = 0.007) and adjusted models (*β* = -0.08, p = 0.003). When evaluating direct effects on support for harm reduction, disease model beliefs about addiction exhibited the strongest inverse association with support for harm reduction (Unadjusted *β* = -0.17, p < 0.001; Adjusted *β* = -0.10, p < 0.001), compared to stigmatizing attitudes toward PWUD (Unadjusted *β* = -0.09, p < 0.001; Adjusted *β* = -0.06, p = 0.003). Conversely, level of familiarity with PWUD was positively associated with support for harm reduction (Unadjusted *β* = 0.06, p < 0.001; Adjusted *β* = 0.07, p < 0.001). Contrary to prediction, we observed no direct association between media consumption and support for harm reduction (Unadjusted *β* = 0.03, p = 0.220; Adjusted *β* = -0.02, p = 0.438). We also assessed whether exposure to media reporting on harm reduction was associated with support for harm reduction via an indirect pathway, i.e., via stigmatizing attitudes. Media exposure exhibited a small, though statistically significant indirect effect on public support for harm reduction via stigma (Unadjusted *β* = 0.01, p *=* 0.026; Adjusted *β* = 0.004, p = 0.048).

## Discussion

To our knowledge, this is the first national study to provide population estimates of public support for harm reduction as a broad approach to substance use, and in relation to seven specific harm reduction interventions. Results indicated that about two-thirds of Canadian adults (64%) were supportive of harm reduction as a general approach to substance use (provincial estimates = 60% - 73%). Importantly, these estimates were obtained using a neutral assessment strategy, i.e. a question that provided a substantive definition in conjunction with popular reasons for support and opposition to harm reduction (i.e., ‘harm reduction refers to public health programs that reduce the harms related to drug use, without requiring people to stop using substances…Supporters think these programs can significantly reduce death and the transmission of disease among people who use drugs, and that these programs can bring them into contact with health and social services that could help in their recovery). Opponents argue that harm reduction programs encourage crime and drug use and should not be offered’). Our results are consistent with previous research that similarly documented substantial public support for harm reduction in select Canadian regions [17, 18, 21, 25, 27]. The present study replicated and extended those findings using survey methods that provided both nationally and provincially representative population estimates. Previous survey research investigated public support only for specific, high-profile harm reduction services (e.g., supervised drug consumption programs) [18]. The present study addressed this limitation by assessing views on a broader range of harm reduction interventions. Our results showed that five of seven interventions were supported by over half of Canadian adults, with strongest support reported for outreach (79%), naloxone (72%), and drug checking (70%), followed by syringe distribution (60%) and supervised injection programs (55%). Two intervention strategies–low threshold opioid agonist treatment and safe inhalation interventions–did not receive majority support in this study. Those results are consistent with CHARPP’s analysis of Canadian newspaper reporting on harm reduction, which demonstrated that these interventions received among the lowest coverage rates over a 17 year period [16].

Taken as a whole, our findings that most Canadian adults support or strongly support harm reduction as an approach to substance use and most harm reduction services could reflect public awareness of Canada’s innovative approach to services for PWUD. Canada is widely recognized as an international leader in harm reduction, starting with early adoption of needle distribution programs in the late 1980s, and more recent implementation of North America’s first supervised drug consumption program in Vancouver in 2003, and North America’s first clinical trial of prescription heroin in 2005 [45, 46]. However, previous CHARPP studies documented relatively weak, rhetorical public policy frameworks governing harm reduction services produced by provincial governments and health authorities [13–15]. Further research is needed to explain this disconnect between inadequate policy supports for harm reduction, despite broad support in the general population. If policy makers are insufficiently aware of such support, they may inadvertently perpetuate a rhetorical, morality policy environment for these services. Cultivating robust knowledge exchange opportunities between governmental and health system decision makers and population and public health researchers investigating determinants of attitudes toward harm reduction services could enhance policy makers’ access to accurate information about public views to support the policy development process.

The second objective of this study was to evaluate a social exposure model predicting public support for harm reduction, drawing on theories in the intergroup relations, addiction, and media communication literatures. Our model hypothesized that three distal variables (personal familiarity with PWUD, disease model beliefs about addiction, and exposure to media coverage on harm reduction) influence support for harm reduction directly, and also indirectly, via their effects on stigmatized attitudes toward PWUD. Overall, our results–which were adjusted for covariates typically considered in this literature (age, political affiliation, income, education)–provided substantial support for the proposed model. As predicted, we observed a significant inverse association between stigmatized attitudes toward PWUD and public support for harm reduction. These results replicate US studies, which similarly reported that stigma toward PWUD appears to undermine public opinion toward harm reduction services [19, 23].

Drawing on the intergroup relations literature, our results also confirmed an inverse association between personal familiarity with PWUD and stigmatized attitudes toward this outgroup, as well as a significant positive association between familiarity and support for harm reduction. These results are consistent with the contact hypothesis, according to which greater exposure to and familiarity with outgroups facilitates empathic personal attitudes [28–30]. One implication of these findings is that efforts to enhance support for harm reduction could focus on programs to strengthen social contact between the public and PWUD.

Eversman [47] notes that “the very nature of addiction and how best to treat it divides harm reduction supporters and opponents” (p. 17), yet to our knowledge, no previous research has examined the role of disease model beliefs about addiction in relation to public support for harm reduction. Results confirmed that greater endorsement of disease model beliefs was associated with greater stigmatized attitudes toward PWUD, as well as lesser support for harm reduction. These findings have not been reported in the literature to our knowledge, and suggest that efforts to enhance support for harm reduction usefully could problematize certain disease model beliefs (e.g. “There are only two possibilities for an alcoholic or drug addict–permanent abstinence or death”).

Finally, drawing on the media communication literature, we also predicted that exposure to media coverage of harm reduction would be positively associated with public support toward these services. Contrary to our prediction, we observed no direct association between media exposure and support for harm reduction. Instead, we observed an indirect effect, such that greater media exposure to harm reduction was associated with lesser stigmatized attitudes toward PWUD, which in turn was associated with greater support for harm reduction. Those results are consistent with cultivation theory [33], according to which exposure to media shapes personal opinions on contentious social issues by altering beliefs about outgroups. Our finding that media exposure to harm reduction was inversely associated with stigmatized attitudes toward PWUD suggests that media can play an important role in promoting support for harm reduction by reducing stigma toward drug users. To that end, media gatekeepers (e.g., editors, content producers) could support efforts to promote positive public views toward harm reduction by prioritizing coverage of PWUD accessing these services who experience positive life changes, thus challenging disease model beliefs about addiction.

### Study limitations

In general, the cross-sectional research design used in this study precludes casual claims as well as assessing directionality. Future research should attempt to replicate our theoretical model using longitudinal study designs. Given that our social exposure model is exploratory in nature, further research is also needed to replicate the specific associations observed in this study: results may change when the measures are administered to populations outside of Canada. Another limitation of the present work is that adults living in the Canadian Territories (Northwest Territories, Nunavut, and Yukon) were not included in the sample. Future research should therefore refine and replicate these methodologies and incorporate those populations. Finally, the constructs in our social exposure model collectively accounted for only 17% of variance in our outcome measure of support for harm reduction. Although we adjusted for political viewpoints, income, education level, and sex of participants, those results imply that there are additional influences on public views not measured in the present research that may also be associated with public support for harm reduction.

## Conclusions

Despite generally favorable opinions toward harm reduction across Canada, weak and rhetorical public policy frameworks currently govern harm reduction services [13, 14]. The present study advances this area beyond past efforts to identify sociodemographic correlates of public views of these contentious services, such as political affiliation, education, or age. Our social exposure model suggests that efforts to change views on these services could focus on problematizing certain disease model beliefs (e.g., “There are only two possibilities for an alcoholic or drug addict–permanent abstinence or death”: a representative item on the disease model beliefs measure used in this study) and creating opportunities to reduce social distance between PWUD, the public, and policy makers.

## Supporting information

S1 File (DOCX)
