# Supplementary material for: Public support for harm reduction: A population survey of Canadian adults
Source: PLoS One. 2021 May 19;16(5):e0251860. doi: 10.1371/journal.pone.0251860 (PMC8133460; doi:10.1371/journal.pone.0251860)
Supplement: S1 File — (DOCX) [file pone.0251860.s001.docx]

**Canadian Harm Reduction Policy Project**

**Public Opinion Survey**

**Screening**

| YEAR/MONTH |
| --- |
| Dropdown |
| What is your date of birth? |
|  |
| YEAR |
| _1910 1910 |
| ... |
| _2015 2015 |
| MONTH |
| _1 January |
| _2 February |
| _3 March |
| _4 April |
| _5 May |
| _6 June |
| _7 July |
| _8 August |
| _9 September |
| _10 October |
| _11 November |
| _12 December |

| RESP_AGE |
| --- |
| Single response |

**[PN: THANK AND TERMINATE IF UNDER 18]**

| RESP_GENDER_ca |
| --- |
| Single response |
| What is your sex? |
|  |
| _1 Male |
| _2 Female |

| QMktSize_CA |
| --- |
| Alphanumeric |
| REQUIRED What is your postal code? (example: A8A8A8) |
| HADD_ZipCode_CA |
| Alphanumeric |
| REQUIRED Postal code: |
|  |
| HCAL_Region1_Label_CA |
| Single Response |
| Hidden Question: Province |
|  |
| (48) Alberta |
| (59) British Columbia |
| (46) Manitoba |
| (13) New Brunswick |
| (10) Newfoundland and Labrador |
| (12) Nova Scotia |
| (61) Northwest Territories |
| (62) Nunavut |
| (35) Ontario |
| (11) Prince Edward Island |
| (24) Quebec |
| (47) Saskatchewan |
| (60) Yukon |
|  |

**[PN: CONTINUE IF AB, BC, MB, NB, NL, NS, ON, PE, QC OR SK, ELSE, THANK AND TERMINATE]**

**Information and Consent**

**Public Attitudes Toward Harm Reduction**

**Background**

Ipsos is conducting a research study on behalf of Dr. Cameron Wild of the University of Alberta. This study aims to describe the attitudes of Canadians toward harm reduction. Results will help the healthcare system study and respond to addictions more effectively.

**What will I be asked to do?**

Ipsos would like to invite you to take part in this survey. If you choose to take part in this study, you will be asked questions about your opinions of harm reduction and your experience of different kinds of substance use. Some of the questions may be about issues that do not apply to you. The survey will take about 15 minutes.

**Do I have to take part?**

Taking part in this survey is your choice. If there are any questions that you do not wish to answer you can select “prefer not to say”, or skip the question. Also, you can end the survey at any time by closing your browser window. If you do not complete the survey, none of your answers will be used. Once you finish the survey, your anonymous answers cannot be withdrawn.

**Privacy and Confidentiality**

Your name and other information will not be linked to the data that is given to the University of Alberta. Your answers to the survey will be kept private. Reports based on this study will only present results in group form. Only the University of Alberta researchers will have access to the survey results. They will store the data in a locked cabinet and on secure servers at the University for 5 years, after which it will be destroyed. All Ipsos Reid survey data is processed and stored on Canadian servers.

**Benefits and Risks**

There may be no direct benefit to you for taking part. In other similar studies, people have not reported experiencing any harm as a result of the kinds of questions we will ask. If you wish to get help or support you can contact a national information service for addiction treatment programs at 1-877-746.1963.

**Scholarly Benefits**

Your participation will directly aid in our understanding of how Canadians respond to substance use.

**Further Information**

The information gathered for this study may be looked at again in the future to help us answer other research questions. If so, an Ethics Board will first review the study to ensure the information will be used in an ethical way.

The Research Ethics Board at the University of Alberta has reviewed this study (Pro00080911) and given it clearance. Should you have any questions or concerns regarding your rights as a participant, or how this study is being conducted, you may contact the University of Alberta's Research Ethics Office at 780-492-2615.  This office has no affiliation with the study investigators. If you have any questions about this study, you can call the Project Coordinator, Jalene Anderson-Baron at 780-492-6753 or 1-866-492-4550.

**[NEW SCREEN]**

**S1. Do you wish to take part in this study?**

Yes

No (please find me another survey)

**[CONTINUE IF YES. THANK & TERMINATE IF NO.]**

**[NEW SCREEN]**

Thank you for your consideration of this invitation.

| 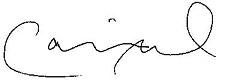 |
| --- |
| Dr. Cameron Wild |
| Professor |
| School of Public Health |
| University of Alberta |
| P. 780.492.6752 E. cam.wild@ualberta.ca |

**MQB**

**Part A**

We’d like to start by asking for your opinion on how Canada is responding to substance use issues.

A1. Law enforcement refers to efforts by the police to stop drug trafficking and use of illegal drugs. In my opinion, Canada is…

*Please select one response only*

Not investing enough in law enforcement to address substance use

Investing just the right amount in law enforcement to address substance use

Investing too much in law enforcement to address substance use

Don’t know/no opinion

Prefer not to say

A2. Treatment includes residential [ ‘rehab’, or other inpatient] programs, detox, and outpatient programs to help people reduce their drug use and start recovery. In my opinion, Canada is…

*Please select one response only*

Not investing enough in treatment for substance use

Investing just the right amount in treatment for substance use

Investing too much in treatment for substance use

Don’t know/no opinion

Prefer not to say

A3. Prevention of substance use includes educational and other programs to stop people from starting to use drugs. In my opinion, Canada is…

*Please select one response only*

Not investing enough in prevention to address substance use

Investing just the right amount in prevention to address substance use

Investing too much in prevention to address substance use

Don’t know/no opinion

Prefer not to say

A4. Harm reduction includes programs like supervised injection sites that are designed to reduce risks and harms of substance use and connect people to healthcare without requiring people to stop using drugs. In my opinion, Canada is…

*Please select one response only*

Not investing enough in harm reduction to address substance use

Investing just the right amount in harm reduction to address substance use

Investing too much in harm reduction to address substance use

Don’t know/no opinion

Prefer not to say

Next, we would like to know your opinions about harm reduction in more detail.

*Harm reduction refers to public health programs that reduce the harms related to drug use, without requiring people to stop using substances*. An example would be providing supervised injection sites to people who inject drugs so that they can use drugs more safely.

There are lots of different opinions about harm reduction. Supporters think these programs can significantly reduce death and the transmission of disease among people who use drugs, and that these programs can bring them into contact with health and social services that could help in their recovery. Opponents argue that harm reduction programs encourage crime and drug use and should not be offered.

A5A. Thinking about media stories you have seen that talk about drug use, have you ever seen or heard media coverage featuring harm reduction?

*Please select one response only*

Yes
No

A5. How much do you support or oppose harm reduction for people who use drugs?

*Please select one response only*

[ACROSS TOP OF GRID]

[ROW 1]

Strongly oppose [ABOVE 1]

Oppose [ABOVE 2]

Don’t know/no opinion [ABOVE 3]

Support [ABOVE 4]

Strongly support [ABOVE 5]

Prefer not to say [ABOVE 6]

[ROW 2]

1

2

3

4

5

6

A6. Please rate your level of agreement with the following statement:

My provincial government should provide more financial and other support to expand harm reduction services

*Please select one response only*

[ACROSS TOP OF GRID]

[ROW 1]

Strongly disagree [ABOVE 1]

Disagree [ABOVE 2]

Don’t know/no opinion [ABOVE 3]

Agree [ABOVE 4]

Strongly agree [ABOVE 5]

Prefer not to say [ABOVE 6]

[ROW 2]

1

2

3

4

5

6

A7. Please rate your level of agreement with the following statement:

The Federal government should provide more financial and other support to expand harm reduction services

*Please select one response only*

[ACROSS TOP OF GRID]

[ROW 1]

Strongly disagree [ABOVE 1]

Disagree [ABOVE 2]

Don’t know/no opinion [ABOVE 3]

Agree [ABOVE 4]

Strongly agree [ABOVE 5]

Prefer not to say [ABOVE 6]

[ROW 2]

1

2

3

4

5

6

**Part B**

In this next section, we ask for your opinions on 7 different harm reduction services.

**[NEW SCREEN]**

Syringe Distribution. This involves the exchange, recovery or distribution of needles or syringes. This may also include the provision of safer injecting supplies (cookers, filters, alcohol swabs, etc.) in addition to needles/syringes. The intended outcome of syringe distribution is to engage people to services and reduce or prevent HIV and other disease transmission, infections, and skin and vein damage.

B1a. How much do you support or oppose syringe distribution for people who use drugs?

*Please select one response only*

[ACROSS TOP OF GRID]

[ROW 1]

Strongly oppose [ABOVE 1]

Oppose [ABOVE 2]

Don’t know/no opinion [ABOVE 3]

Support [ABOVE 4]

Strongly support [ABOVE 5]

Prefer not to say [ABOVE 6]

[ROW 2]

1

2

3

4

5

6

B1b. Please rate your level of agreement with the following statements about syringe distribution.

*Please select one response for each item*

[ACROSS TOP OF GRID]

[ROW 1]

Strongly disagree [ABOVE 1]

Disagree [ABOVE 2]

Don’t know/no opinion [ABOVE 3]

Agree [ABOVE 4]

Strongly agree [ABOVE 5]

Prefer not to say [ABOVE 6]

[ROW 2]

1

2

3

4

5

6

[DOWN SIDE OF GRID – RANDOMIZE ORDER]

Syringe distribution encourages drug use

Syringe distribution increases the safety of people who use drugs

Syringe distribution increases crime in the community

Syringe distribution connects people who use drugs to other health and social services

Naloxone. Naloxone is a medication that reverses the effects of opioid overdose, similar to an Epipen that reverses an anaphylactic allergic reaction. Naloxone is the generic name of the drug, which is sometimes sold under the brand name Narcan in Canada. Take home naloxone kits are distributed in various settings, including harm reduction programs, pharmacies, treatment programs, and hospitals, and enable lay persons to intervene in the event of an overdose.

B2a. How much do you support or oppose distributing naloxone kits in the community/?

*Please select one response only*

[ACROSS TOP OF GRID]

[ROW 1]

Strongly oppose [ABOVE 1]

Oppose [ABOVE 2]

Don’t know/no opinion [ABOVE 3]

Support [ABOVE 4]

Strongly support [ABOVE 5]

Prefer not to say [ABOVE 6]

[ROW 2]

1

2

3

4

5

6

B2b. Please rate your level of agreement with the following statements about naloxone.

*Please select one response for each item*

[ACROSS TOP OF GRID]

[ROW 1]

Strongly disagree [ABOVE 1]

Disagree [ABOVE 2]

Don’t know/no opinion [ABOVE 3]

Agree [ABOVE 4]

Strongly agree [ABOVE 5]

Prefer not to say [ABOVE 6]

[ROW 2]

1

2

3

4

5

6

[DOWN SIDE OF GRID – RANDOMIZE ORDER]

Naloxone encourages drug use

Naloxone increases the safety of people who use drugs

Naloxone increases crime in the community

Naloxone connects people who use drugs to other health and social services

Supervised Consumption Services. Interventions designed to reduce individual and community impacts associated with injection and non-injection drug use. Supervised Injection Facilities are an example of this intervention. These are legally sanctioned, medically supervised spaces that provide people who inject their own personally acquired drugs in a hygienic environment under the supervision of healthcare professionals. Emergency medical assistance is provided in the event of an overdose.

B3a. How much do you support or oppose supervised consumption services for people who use drugs?

*Please select one response only*

[ACROSS TOP OF GRID]

[ROW 1]

Strongly oppose [ABOVE 1]

Oppose [ABOVE 2]

Don’t know/no opinion [ABOVE 3]

Support [ABOVE 4]

Strongly support [ABOVE 5]

Prefer not to say [ABOVE 6]

[ROW 2]

1

2

3

4

5

6

B3b. Please rate your level of agreement with the following statements about supervised consumption.

*Please select one response for each item*

[ACROSS TOP OF GRID]

[ROW 1]

Strongly disagree [ABOVE 1]

Disagree [ABOVE 2]

Don’t know/no opinion [ABOVE 3]

Agree [ABOVE 4]

Strongly agree [ABOVE 5]

Prefer not to say [ABOVE 6]

[ROW 2]

1

2

3

4

5

6

[DOWN SIDE OF GRID – RANDOMIZE ORDER]

Supervised consumption encourages drug use

Supervised consumption increases the safety of people who use drugs

Supervised consumption increases crime in the community

Supervised consumption connects people who use drugs to other health and social services

Low Threshold Opioid Treatment. This intervention is designed to reduce the risks associated with using opioids, in which opioid agonist medication (i.e., methadone, suboxone) is delivered in a low-threshold setting. A low-threshold setting is one that does not impose/require abstinence as a condition for services, and attempts to reduce other barriers associated with accessing services, such as a lack of ID.

B4a. How much do you support or oppose low threshold opioid substitution for people who use drugs?

*Please select one response only*

[ACROSS TOP OF GRID]

[ROW 1]

Strongly oppose [ABOVE 1]

Oppose [ABOVE 2]

Don’t know/no opinion [ABOVE 3]

Support [ABOVE 4]

Strongly support [ABOVE 5]

Prefer not to say [ABOVE 6]

[ROW 2]

1

2

3

4

5

6

B4b. Please rate your level of agreement with the following statements about low threshold opioid substitution.

*Please select one response for each item*

[ACROSS TOP OF GRID]

[ROW 1]

Strongly disagree [ABOVE 1]

Disagree [ABOVE 2]

Don’t know/no opinion [ABOVE 3]

Agree [ABOVE 4]

Strongly agree [ABOVE 5]

Prefer not to say [ABOVE 6]

[ROW 2]

1

2

3

4

5

6

[DOWN SIDE OF GRID – RANDOMIZE ORDER]

Low threshold opioid substitution encourages drug use

Low threshold opioid substitution increases the safety of people who use drugs

Low threshold opioid substitution increases crime in the community

Low threshold opioid substitution connects people who use drugs to other health and social services

Community Outreach. Intervention designed to engage people experiencing marginalization, disconnection or alienation from mainstream and/or targeted services and supports, in which education, harm reduction supplies and care are delivered outside of a fixed location (i.e. mobile van).

B5a. How much do you support or oppose outreach for people who use drugs?

*Please select one response only*

[ACROSS TOP OF GRID]

[ROW 1]

Strongly oppose [ABOVE 1]

Oppose [ABOVE 2]

Don’t know/no opinion [ABOVE 3]

Support [ABOVE 4]

Strongly support [ABOVE 5]

Prefer not to say [ABOVE 6]

[ROW 2]

1

2

3

4

5

6

B5b. Please rate your level of agreement with the following statements about outreach.

*Please select one response for each item*

[ACROSS TOP OF GRID]

[ROW 1]

Strongly disagree [ABOVE 1]

Disagree [ABOVE 2]

Don’t know/no opinion [ABOVE 3]

Agree [ABOVE 4]

Strongly agree [ABOVE 5]

Prefer not to say [ABOVE 6]

[ROW 2]

1

2

3

4

5

6

[DOWN SIDE OF GRID – RANDOMIZE ORDER]

Outreach encourages drug use

Outreach increases the safety of people who use drugs

Outreach increases crime in the community

Outreach connects people who use drugs to other health and social services

Drug Checking. Testing illegal drugs to inform consumers about the content. The idea is to potentially reduce harm from ingesting unknown or contaminated substances or more dangerous substances than intended. This intervention can be applied through various methods, ranging from do-it-yourself pill testing kits to more rigorous testing using lab equipment.

B6a. How much do you support or oppose drug checking for people who use drugs?

*Please select one response only*

[ACROSS TOP OF GRID]

[ROW 1]

Strongly oppose [ABOVE 1]

Oppose [ABOVE 2]

Don’t know/no opinion [ABOVE 3]

Support [ABOVE 4]

Strongly support [ABOVE 5]

Prefer not to say [ABOVE 6]

[ROW 2]

1

2

3

4

5

6

B6b. Please rate your level of agreement with the following statements about drug checking.

*Please select one response for each item*

[ACROSS TOP OF GRID]

[ROW 1]

Strongly disagree [ABOVE 1]

Disagree [ABOVE 2]

Don’t know/no opinion [ABOVE 3]

Agree [ABOVE 4]

Strongly agree [ABOVE 5]

Prefer not to say [ABOVE 6]

[ROW 2]

1

2

3

4

5

6

[DOWN SIDE OF GRID – RANDOMIZE ORDER]

Drug checking encourages drug use

Drug checking increases the safety of people who use drugs

Drug checking increases crime in the community

Drug checking connects people who use drugs to other health and social services

Safer Inhalation Kits. This intervention is designed to reduce risk associated with smoking drugs in which safer smoking equipment (stems, mouthpieces, screens, push sticks, pipes, etc.) are made available and distributed without requiring the return/exchange of used equipment.

B7a. How much do you support or oppose safer inhalation kits for people who use drugs?

*Please select one response only*

[ACROSS TOP OF GRID]

[ROW 1]

Strongly oppose [ABOVE 1]

Oppose [ABOVE 2]

Don’t know/no opinion [ABOVE 3]

Support [ABOVE 4]

Strongly support [ABOVE 5]

Prefer not to say [ABOVE 6]

[ROW 2]

1

2

3

4

5

6

B7b. Please rate your level of agreement with the following statements about safer inhalation kits.

*Please select one response for each item*

[ACROSS TOP OF GRID]

[ROW 1]

Strongly disagree [ABOVE 1]

Disagree [ABOVE 2]

Don’t know/no opinion [ABOVE 3]

Agree [ABOVE 4]

Strongly agree [ABOVE 5]

Prefer not to say [ABOVE 6]

[ROW 2]

1

2

3

4

5

6

[DOWN SIDE OF GRID – RANDOMIZE ORDER]

Safer inhalation kits encourage drug use

Safer inhalation kits increase the safety of people who use drugs

Safer inhalation kits increase crime in the community

Safer inhalation kits connect people who use drugs to other health and social services

**Part C**

This next set of questions asks about your personal experiences and opinions about substance use. Remember, there are no right or wrong answers, and your responses are completely confidential.

C1. Please read each of the following statements carefully. After you have read all of the statements below, place a check by every statement that represents your personal experience with persons with a substance use problem.

I have watched a movie or television show in which a character depicted a person with a substance use problem.

My job involves providing services/treatment for persons with a substance use problem.

I have observed, in passing, a person I believe may have had a substance use problem.

I have observed persons with a substance use problem on a frequent basis.

I have a substance use problem.

I have worked with a person who had a substance use problem at my place of employment.

I have never observed a person that I was aware had a substance use problem.

A friend of the family has a substance use problem.

I have a relative who has a substance use problem.

I have watched a documentary on television about substance use problems.

I live with a person who has a substance use problem.

None of the above

C2. Thinking about media stories you have seen that talk about drug use, have you ever seen or heard media coverage featuring mothers who have had a child die from a fatal drug overdose?

*Please select one response only*

Yes
No

C3. Which of the following statements most closely matches your personal definition of recovery in terms of substance use?

*Please select one response only*

No use of any substance – drugs or alcohol

No use of any substance – drug or alcohol – except as prescribed by your doctor

No use of substance of choice but some use of other substances

Moderate or controlled use of any substance – drug or alcohol

Moderate or controlled use of alcohol

Moderate or controlled use of drugs

Don’t know/no opinion

Prefer not to say

C4. This next set of items asks about your personal opinions about people who have a substance use problem.

*Please select one response for each item*

[ACROSS TOP OF GRID]

[ROW 1]

Definitely not [ABOVE 1]

Probably not [ABOVE 2]

Not sure/don’t know [ABOVE 3]

Probably [ABOVE 4]

Definitely [ABOVE 5]

Prefer not to say [ABOVE 6]

[ROW 2]

1

2

3

4

5

6

[DOWN SIDE OF GRID. RANDOMIZE ORDER.]

Would you be afraid to talk to someone who has a substance use problem?

Would you be upset or disturbed to be in the same room with someone who has a substance use problem?

Would you make friends with someone who has a substance use problem?

Would you feel embarrassed or ashamed if your friends knew that someone in your family has a substance use problem?

C5. We’d like to know how much you agree or disagree with these statements about how people who have problems with alcohol and other drugs. Remember, there are no right or wrong answers; we are interested in your opinion.

*Please select one response for each item*

[ACROSS TOP OF GRID]

[ROW 1]

Strongly disagree [ABOVE 1]

Somewhat disagree [ABOVE 2]

Neither disagree nor agree [ABOVE 3]

Somewhat agree [ABOVE 4]

Strongly agree [ABOVE 5]

[ROW 2]

1

2

3

4

5

[DOWN SIDE OF GRID. RANDOMIZE ORDER.]

Every alcoholic and addict must accept that he or she is powerless over alcohol and drugs, and can never

drink or use drugs again

Daily use of small amounts of substances like marijuana is not necessarily harmful

Every alcoholic or addict is one drink or one hit away from a total relapse

The society or culture in which one grows up has a significant influence on whether he or she becomes

an alcoholic or addict

Marijuana is accepted in some communities, so there is nothing wrong with using it while there

A person’s environment plays an important role in determining whether he or she develops alcoholism
or drug addiction

Once a person is an alcoholic or an addict, he or she will always be an alcoholic or an addict

Personal use of drugs should be legal in the confines of one’s own home

Alcoholism and drug addiction are caused, in part, by growing up in a dysfunctional family

Usually if alcoholics and addicts fail to recover in Alcoholics Anonymous (AA), Narcotics Anonymous (NA) or treatment, it is because they are unmotivated and in denial

As long as no one else is harmed, people should have the right to engage in whatever behaviours they

want

If an alcoholic or addict is sober or straight for five years, then starts drinking or using drugs again, he or

she is right back where he or she left off in the development of the disease

Alcoholism and drug addiction are caused, in part, by what one learns about alcohol and drugs and the

drinking/drug use patterns of one’s family and peers

Some people use drugs, but never become addicted

A person can develop alcoholism or drug addiction because of underlying psychological problems

There are only two possibilities for an alcoholic or drug addict – permanent abstinence or death

Addiction does not always result in a negative outcome

If an alcoholic has a drink, or if an addict takes a hit, they lose control and are unable to stop from

getting drunk or high

People fail to consider that addictive behaviours may be positive

People often outgrow drug and alcohol addiction

There are people who have significant problems with alcohol, but who are not alcoholics

Addicts can learn to control their use

**Part D**

This last set of questions asks more general information about you.

D1. For each of the following statements, please indicate your level of agreement

*Please select one response for each item*

[ACROSS TOP OF GRID]

[ROW 1]

Strongly disagree [ABOVE 1]

Disagree [ABOVE 2]

Don’t know/no opinion [ABOVE 3]

Agree [ABOVE 4]

Strongly agree [ABOVE 5]

Prefer not to say [ABOVE 6]

[ROW 2]

1

2

3

4

5

6

[DOWN SIDE OF GRID. RANDOMIZE ORDER.]

The government should increase its assistance for the poor

The government should lower taxes

The government should be actively involved in solving problems that develop in society

The government has taken over too many things that should be handled by individuals, families, and private businesses

D2. Please indicate how you would describe your political views

*Please select one response only*

Very liberal

Mostly liberal

Equally liberal and conservative

Mostly conservative

Very conservative

I don’t have any political views

Prefer not to say

D3. What is your annual household income?

*Please select one response only*

<$50,000

$50,000 - $100,000

>$100,000

Prefer not to say

D4. What is your level of education?

*Please select one response only*

High school diploma or less

College or Technical school

University graduate

D5. Do you live in an urban or rural setting?

*Please select one response only*

Urban

Rural
